# Layer-by-Layer Encapsulation of Herbicide-Degrading Bacteria for Improved Surface Properties and Compatibility in Soils

**DOI:** 10.3390/polym13213814

**Published:** 2021-11-04

**Authors:** Reut Gal, Neriya Perez-Lapid, Yael Zvulunov, Adi Radian

**Affiliations:** Faculty of Civil and Environmental Engineering, Technion—Israel Institute of Technology, Technion City, Haifa 32000, Israel; kupgal17@gmail.com (R.G.); neriya@campus.technion.ac.il (N.P.-L.); syaelzv@campus.technion.ac.il (Y.Z.)

**Keywords:** polyelectrolytes, polydiallyldimethylammonium chloride, layer-by-layer (LbL), biodegradation, herbicides, pollution, atrazine

## Abstract

*E. coli* cells overexpressing the enzyme atrazine chlorohydrolase were coated using layer-by-layer self-assembly. The polymeric coating was designed to improve the surface properties of the cells and create positively charged, ecologically safe, bio-hybrid capsules that can efficiently degrade the herbicide atrazine in soils. The physio-chemical properties of the bacteria/polymer interface were studied as a function of the polymeric composition of the shell and its thickness. Characterization of cell viability, enzyme activity, morphology, and size of the bio-capsules was done using fluorescence spectroscopy, BET and zeta potential measurements and electron microscopy imaging. Out of several polyelectrolytes, the combination of polydiallyldimethylammonium chloride and polysodium 4-styrenesulfonate improved the surface properties and activity of the cells to the greatest extent. The resulting bio-hybrid capsules were stable, well-dispersed, with a net positive charge and a large surface area compared to the uncoated bacteria. These non-viable, bio-hybrid capsules also exhibited a kinetic advantage in comparison with uncoated cells. When added to soils, they exhibited continuous activity over a six-week period and atrazine concentrations declined by 84%. Thus, the concept of layer-by-layer coated bacteria is a promising avenue for the design of new and sustainable bioremediation and biocatalytic platforms.

## 1. Introduction

In situ bioremediation through bioaugmentation with an inoculum of a pure or mixed culture could be highly efficient and environmentally safe remediation method [1], however, the introduction of non-indigenous species into soil could also pose ecological stress, and the use of genetically modified organisms (GMOs) is often regulatorily restricted [2,3]. Catalytic agents for field bioremediation must be stable, anchored to soil particles to avoid being leached out and active under various physical and chemical environmental conditions. While some bioremediation enzymes can transform toxic compounds into less harmful metabolites, most enzymes are not applicable as biocatalysts outside of cells or fail to retain their biodegrading activities for an adequate time span. Given the time-consuming practices and relatively high overall cost associated with the production and purification of enzymes, alternative bioremediating tools that are inexpensive, robust, and environmentally responsible are in high demand [4,5].

In the last three decades, the layer-by-layer (LbL) self-assembly methods have been widely used to synthesize various functional cell-based polymeric capsules [6,7]. This technology relies on different types of intermolecular interactions between the cell membrane and the polymers, through a rapid self-assembly process. It does not require elaborate synthesis nor is it based on toxic materials. The thickness of the LbL shells is tunable at the nanometer scale by adjusting the number of layers, while the environment within the shell remains controllable and steady over time. To date, cell encapsulation via LbL has been mainly utilized in cutting-edge biomedical fields such as cell-based biosensors, cell transplantation, cell/molecule delivery, and tissue engineering [7]. However, in recent years, bio-polymeric capsules have extended far beyond their scope in biomedicine, and into the area of environmental science [6,8,9,10,11].

Pollutant removal strategies using LbL technologies have been previously demonstrated, highlighting the ease of use, stability, and reusability of LbL capsules [12,13,14,15]. Most of the recent advances in LbL technologies for environmental purposes have been established in membrane design and synthesis [11,14,16,17], while LbL-coating of bacteria for remediating purposes has not been widely explored [12,18,19,20]. Sakkos et al. [20], used a LbL coating based on a quaternary ammonium polyelectrolyte and silicon alkoxide (PolyDADMAC/silica) to create an exoskeleton which immobilized, protected, and enhanced the activity of their chosen bacteria. They observed that the coating protected the cells from desiccation, freeze/thaw, high temperatures, osmotic shock, enzymatic attack by lysozyme, and predation by protozoa. Deng et al. [18], designed biochar-based LbL alginate/chitosan microcapsules for the bioremediation of phenanthrene from polluted soil. Application of the capsules improved phenanthrene removal in comparison with the control (80% vs. 62% removal) and enhanced the biodegrading microbial community in the soil. Applying LbL techniques is therefore highly attractive for improving bioremediation platforms, as it provides a well-controlled microenvironment for GMOs and mitigates the ecological risks posed by the addition of non-indigenous microbial population into contaminated sites [21]. It also protects the remediating agents within from extreme soil conditions and natural predation, which can hinder bacterial growth or limit the enzymes’ robustness.

In the present work, LbL assembly was applied to atrazine (herbicide)-degrading bacteria to enhance biodegradation, while creating positively charged, high surface area and non-viable bioparticles, that can be well retained in soils. Atrazine (ATZ) is a toxic endocrine disruptor according to World Health Organization (WHO), with high persistence in soil and water sources. It has a maximum contaminant level (MCL) in drinking water of 3 µg/L. However, its metabolite hydroxyatrazine (OH-ATZ), does not have any regulations regarding its amount in drinking water, nor any neuroendocrine-disrupting properties; it is not considered a product of concern by WHO [22]. The enzyme atrazine chlorohydrolase (AtzA) is responsible for the dechlorination of ATZ, leading to the formation of OH-ATZ. Studies by Wackett and his group have provided ample evidence to the advantages of using *E. coli* overexpressing AtzA for reaching higher degradation rates than typically observed in native bacteria both in the lab and in the field [23,24,25]. Nevertheless, drawbacks related to limited bioavailability caused by low concentrations of the pollutant, sustainability over time and the use of GMO remain.

**Figure 1:**
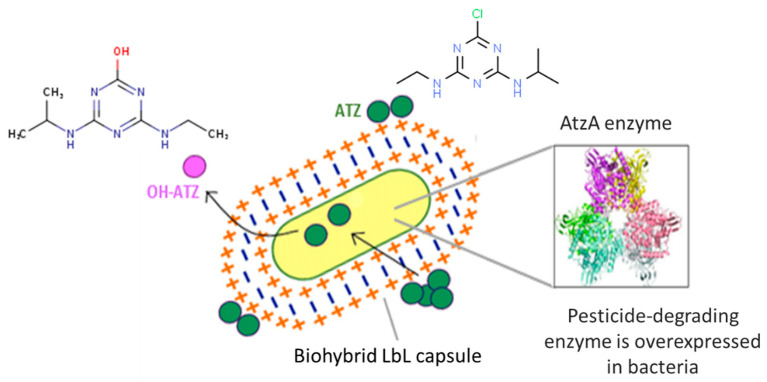
Illustration of the biohybrid LbL capsule based on alternating, oppositely charged polyelectrolytes, coating bacteria overexpressing the enzyme AtzA.

Herein, we fabricated biohybrid LbL capsules, designed for an improved, environmentally safe, and continuous remediation regime (Figure 1). As a proof of concept, we coated biodegrading bacteria, able to transform ATZ into OH-ATZ, via the LbL technique using several polyelectrolytes. In-depth characterization of the coated surface of the cells was done using BET, fluorescent and zeta potential measurements and electron microscopy imaging. Activity and kinetics of ATZ degradation were tested in batch experiments and in soil microcosms. We believe that such confinement of microbial cells within layers of polyelectrolytic matrix can allow for the physical isolation of cells from the external environment and hinder undesired proliferation while maintaining a favorable internal micro-environment in the bacterial cell.

## 2. Materials and Methods

### 2.1. Materials

*E. coli* DH5α (pMD4) were kindly received from Prof. Wackett from the Biotechnology Department at the University of Minnesota (Minneapolis, MN, USA). Poly(sodium 4-styrenesulfonate) (PSS, Mw = 70 kDa), poly(dimethyldiallylammonium chloride) (PolyDADMAC, Mw = 100–200 kDa), chitosan (Mw = 50–190 kDa, ≥75% deacetylation degree) and poly-4-vinylpyridine-*co*-styrene (PVPcoS, Mw = 60 kDa) were obtained from Sigma-Aldrich (Jerusalem, Israel). PSS and PolyDADMAC were dissolved in deionized water (DIW), reaching a final solution of 2 mg/mL, at neutral pH. Chitosan was dissolved in 0.1% acetic acid then pH was adjusted to 5 with 0.1 M NaCl, reaching a final solution of 2 mg∙mL^−1^. PVPcoS was dissolved in a 6.5 mM sulfuric acid solution, resulting in a total of 45% charged monomers, producing H-PVPcoS in a final solution of 3 mg∙mL^−1^ [26]. All reagents used for high pressure liquid chromatography (HPLC) were purchased from Sigma-Aldrich. ATZ (94% technical grade) was acquired from Agan Chemicals (Ashdod, Israel). ATZ stock solution (0.05 millimolar) was prepared in methanol and stored at −16 °C in a freezer.

### 2.2. Bacterial Cultures

*E. coli* cells were taken from a frozen stock (stored at −80 °C), streaked on an agar plate supplemented with 30 µg∙mL^−1^ chloramphenicol, and incubated overnight at 37 °C. All plates were stored at 4 °C for up to 1 month. Precultures of bacteria were made by inoculating a single bacterial colony in 5 mL Luria-Bertani medium and incubated at 37 °C, shaken at 250 revolutions per minute (RPM) for 6 h. Intermediate cultures were grown by inoculation with 1% (*v*/*v*) starter culture into Erlenmeyer flask containing superbroth medium: [24] 1.2% tryptone (*w*/*v*), 1.4% yeast extract (*w*/*v*), 0.5% glycerol (*v*/*v*), 0.38% (*w*/*v*) monobasic potassium phosphate, and 1.25% (*w*/*v*) dibasic-potassium phosphate, supplemented with 30 μg∙mL^−1^ chloramphenicol (at pH 7.4), and grown at 37 °C with vigorous agitation for 18 h. Bacteria were harvested by centrifugation at 6000 RPM for 20 min (min) at 4 °C and resuspended in potassium-phosphate buffer (PBS) (0.1 M, pH 7.4) to a final concentration of 1 g of cell∙mL^−1^.

### 2.3. Encapsulation of Bacteria by LbL Self-Assembly

Biohybrid capsules were fabricated by using the LbL assembly technique [27,28], which is performed through the alternate deposition of oppositely charged polyelectrolytes onto the cells. Fabrication process started by adding 150 µL resuspended cells (in PBS) to 1.5 mL polycation solution (either polyDADMAC, chitosan or H-PVP-co-S) in 2 mL Eppendorf tubes. Polycations were allowed to adsorb for 10 min under constant agitation in room temperature. Tubes were then lightly centrifuged (6000 RPM for 5 min), the supernatant was discarded, and 3 washing steps using PBS were followed. One tube was resuspended DIW and set to the side to determine the ζ-potential in Zetasizer Nano ZS (Malvern Instruments, Malvern, UK) at 25 °C. If charge reversal was confirmed, all other tubes were then added to a PSS solution. This process was repeated a maximum of seven cycles, reaching one, three, five or seven layers. ζ-potentials reported in this work are an average of ten measurements each.

### 2.4. Characterization of Biohybrid LbL Capsules

Size analysis: Mean particle size diameter and polydispersity index (PDI) of coated or uncoated bacterial cell suspensions were detected using dynamic light scattering (DLS) and measured using a Zetasizer Nano ZS. The specific surface areas, Brunauer-Emmett-Teller (BET), and nitrogen sorption isotherms were obtained using a surface area analyzer (NOVAtouch LX4, Quantachrome, Boynton Beach, FL, USA) at liquid nitrogen temperature (77.35 K), with adsorption branch from P/P_0_ = 0.02–0.99. 300–400 milligrams of dried LbL coated cells or free cells (FCs) were degassed under a nitrogen atmosphere at 80 °C for 6 h prior to the analysis.

Morphological analysis: images were taken by scanning electron microscopy (SEM, Ultra-Plus High-Resolution SEM, Zeiss, Oberkochen, Germany) with an acceleration voltage of 1.5–2.5 kV, and transmission electron microscopy (TEM, Talos L120C, Thermo-Scientific, Waltham, MA, USA) with an acceleration voltage of 120 kV. The samples were prepared in a 1 mL solution of coated or uncoated cells, centrifuged for 5 min and resuspended in 1 mL fixative (2.5% glutaraldehyde (GA) in PBS buffer (0.1 M, pH 7.4) for 1 h at room temperature for SEM, and 2% paraformaldehyde and 2% GA in 0.1 M cacodylate buffer for 90 min at room temperature for TEM). After fixation, a few microliters of cells were dropped onto silicon chips for SEM, washed with PBS, and dehydrated through several washes in a gradually increasing ethanol solutions (30, 50,70, 80, 90 and 100% ethanol, 15 min each). Samples were then left to dry overnight and sputter-coated with carbon. For TEM, cells were washed with cacodylate buffer and embedded in agarose. Following the wash with cacodylate buffer, the samples were postfixed for 1 h on ice with 2% osmium tetra oxide, block stained with 1% uranyl acetate and dehydrated using graded ethanol series. Finally, the samples were embedded in Epon812 and polymerized for 24 h at 60°C. Thin 75 nm sections were cut using a UC7 ultramicrotome (Leica, Wetzlar, Germany) and post-stained with 2% uranyl acetate.

Cell viability was quantified using a live/dead cell viability assay, performed with propidium iodide (PI, Mw of 668). A solution of 0.1 mg∙mL^−1^ of the LbL coated bacteria (1, 3, 5 and 7 layers) and FCs was doped with 2 µL of a 20 mM solution of PI in dimethyl sulfoxide. The consequent fluorescence of the doped cells was quantified by a fluorescence spectrometer (FS-2, SCINCO, Seoul, Korea), at excitation and emission wavelengths of 535 nm and 619 nm, respectively. All measurements were conducted in a quartz cuvette at 24 °C.

Viable count method: encapsulated cells or FCs were plated on selective agar plates supplemented with 25 mg∙mL^−1^ chloramphenicol, to examine formation of colonies. Inoculation of cells was processed right after the LbL depositing procedure or after incubation in soil for 1 week, 2 weeks and 6 weeks. The plates were incubated at 37 °C for 1 day.

### 2.5. ATZ Biodegradation Activity Assays

Activity measurements of coated and uncoated cells were performed at room temperature in 2 mL Eppendorf tubes under constant agitation, for 20 min. The reaction was initiated by adding 15 μM atrazine in PBS to the resuspended cells (final bacterial concentration of 2.5 mg/mL). Enzymatic reaction was terminated by immersing the Eppendorf tubes in a water bath heated to 95 °C for 10 min. Samples were then filtered through 0.22 μm pore size polytetrafluoroethylene syringe filters and placed in HPLC vials. HPLC measurements were carried out on an 1260 Infinity series HPLC system (Agilent Technologies, Santa Clara, CA, USA) equipped with a photodiode array detector. An analytical C_18_ reversed phase Agilent column was used (25 cm × 4.6 mm I.D.), with a wavelength of 220 nm selected for all measurements. The derivatives were eluted with methanol and water (65:35, *v*/*v*) at a flow rate of 1 mL∙min^−1^.

Kinetic experiments were carried out at a fixed concentration (15 µM) at allotted times or at fixed time (20 min) with varying concentrations. The relationship between OH-ATZ formation and initial concentration of ATZ was described with Michaelis–Menten (MM) kinetics model. The Michaelis-Menten equation for this system is:

$$v=\frac{V_{max}\left[S\right]}{K_{M}+\;\left[S\right]}$$

where *V_max_* is the maximum velocity at maximum substrate [*S*] concentration, and *K_M_* is the substrate concentration at which the reaction velocity is 50% of *V_max_* [29]. The fitting was preformed using the Origin software (vs. 9.7, OriginLab Corporation, North Hampton, MA, USA).

### 2.6. Removal of ATZ from Soil

Soil sample collection and bacterial incubation: two different types of soil were selected; (1) Grumusol—coarse grained soil with high clay content (montmorillonite >59%). (2) Brown-red Loam—sandy soil with low clay content (7%). Soil samples were taken from northern Israel, from the upper 30 cm of the profiles, air–dried and sieved through a 2-mm mesh. Shortly before the experiments, soil samples were autoclaved and hydrated with autoclaved DIW, then stored in closed buckets at room conditions throughout the period of experiment. Soil inoculation experiments were conducted in 50-mL screw cap centrifuge tubes containing 10 g of soil (20% moisture), with added bacteria in a concentration of 30 mg∙mL^−1^ in PBS. ATZ biodegradation experiment in soil was initiated by adding 2 mL of 15 µM ATZ in PBS with constant agitation at room temperature for 20 min, following similar reaction termination and filtration as in the solution experiments.

To ensure proper extraction of ATZ/OH-ATZ from soil, 15 mL of 100% MeOH was added to soil samples, prior to incubation for 24 h at 37 °C under constant agitation. Samples were then centrifuged, and supernatant was collected, filtered, and analyzed by HPLC.

### 2.7. Statistical Analysis

It should be noted that biological activity may vary slightly between bacteria grown on different days due to different amounts of expressed proteins. To account for these differences, each experiment was performed with three replicates and all treatments were performed on the same batch of bacteria. The results indicate the means ± standard deviation (SD). Differences between groups were compared by *t*-test: two-sample assuming unequal variances (*p* < 0.05), using the Excel software (Microsoft, Redmond, WA, USA).

## 3. Results

ATZ degrading bacteria were chosen as a model for active and efficient herbicide-biodegrading agents. Several polyelectrolytes were chosen for the LbL coating based on previous findings describing ATZ adsorption, biocompatibility, low-cost, toxicity, and ecological safety: PSS-an anionic, low-cost, non-toxic polymer with hydrophobic functional groups [30,31]. PolyDADMAC-a highly positively charged polymer with tertiary amine groups, chosen due to its wide application as a safe coagulant in drinking water remediation technologies [32]. HPVP-*co*-S, a copolymer composed of benzene and pyridine rings at a ratio of 1:9, in which only 40% of the monomers were charged, was chosen due to its previously establish ATZ adsorption capability [26]. And chitosan, a high molecular-weight linear polymer, which is biodegradable and has also been shown to adsorb ATZ [33].

### 3.1. Synthesis of Biohybrid LbL Capsules

**Figure 2:**
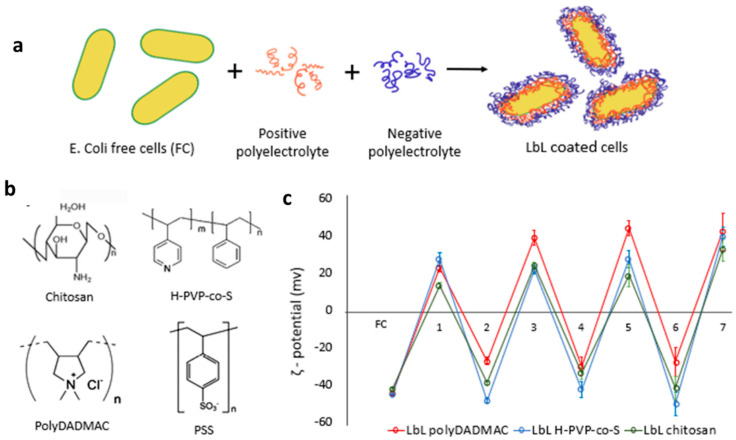
(**a**) Illustration of the LbL deposition of polyelectrolytes. (**b**) Chemical structure of the polyelectrolytes used in this study. (**c**) ζ potential measurements showing charge reversal after each layer added.

The LbL coated bacteria were fabricated by the self-assembly of polyelectrolytes as shown in Figure 2a. At pH 7.2, the surface of uncoated *E. coli* cells carries a net negative charge of −44.77 mV ± 1.3 (Figure 2c). Adsorption of the first layer of polycation resulted in charge reversal of the surface. Surface charge alternation with sequential deposition of polycation and polyanion layers was observed for all polyelectrolyte combinations: PolyDADMAC/PSS, chitosan/PSS, and H-PVP-*co*-S/PSS, confirming that the assembly took place on the surface.

### 3.2. Biodegradation of ATZ by the Biohybrid LbL Capsules

**Figure 3:**
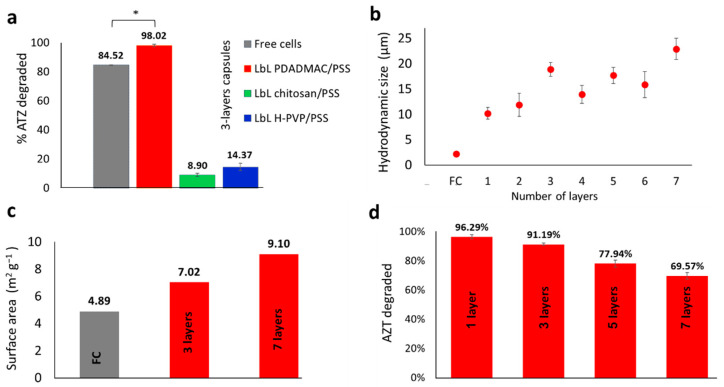
(**a**) ATZ degradation by FC and 3 different LbL biohybrid capsules at initial ATZ oncentration of 15µM (**b**) Hydrodynamic size of FCs and LbL polyDADMAC/PSS biohybrid capsules (**c**) Specific surface area of FC and LbL polyDADMAC/PSS biohybrid capsules (**d**) ATZ degradation by LbL polyDADMAC/PSS biohybrid capsules as a function of number of layers. * Indicates significant difference between the groups (*p* < 0.05).

All capsules composed out of the different polycations along with PSS exhibited a high positive ζ-potential, associated with the formation of stable complexes, which repel each other in colloidal dispersions and have a minor tendency to aggregate [34]. The ability of the LbL coated bacteria to transform ATZ into OH-ATZ improved significantly (*p* < 0.05) compared to free cells (FC) only when the bacteria were coated with polyDADMAC/PSS (Figure 3a). The other capsules, based on chitosan or HPVP-*co*-S, seemed to hinder ATZ biodegradation. It should be noted that FC controls were subjected to the same treatment steps as encapsulated cells, including centrifugation cycles, rinsing steps and resuspensions in PBS, however the results were not significantly different than fresh free cells not subjected to the same treatment regime.

The difference in the biodegradation ability observed for polyDADMAC/PSS coated microbes in comparison to the other polyelectrolytes could be attributed to increased diffusion of ATZ intercellularly, due to: (i) increased surface area of the LbL bacteria capsule, and/or (ii) increased diffusion into the bacterial membrane. We suggest the higher charge density of polyDADMAC compared to chitosan or HPVP-*co*-S can be the controlling parameter—effecting LbL growth patterns, coating configuration and membrane interactions.

To examine the LbL coating configuration, BET and size measurements were performed on polyDADMAC/PSS coated cells and compared to FC. As seen in Figure 3b, FC exhibited the smallest diameter and the hydrodynamic diameter of polyDADMAC/PSS coated cells increased as more layers were added to the LbL structure. However, specific surface area analysis performed by BET showed a higher surface area for coated cells, increasing from 4.89 m^2^∙g^−1^ for uncoated cells, reaching 7.02 m^2^∙g^−1^ for the 3-layers LbL capsule and 9.1 m^2^∙g^−1^ for the 7-layers capsule (Figure 3c). The increased surface area attributed to the polyelectrolytic layers is postulated to increase the flux of ATZ and its metabolite OH-ATZ in and out of the cells, increasing ATZ transformation rates. The data obtained from DLS and BET measurements show counterintuitive results, as surface to volume ratio should decrease as size increases. This could be explained by the arrangement of the PSS/PolyDADMAC layers on the membrane surface (loops and tails) [35,36,37]. The larger surface area of the capsules compared to FC is also speculated to aid the problem of limited bioavailability. Often the pesticide concentrations in soils are too low for the bacteria to efficiently degrade it, resulting in a slow process of several months. The physicochemical properties of contaminated soils are known to dramatically affect the bioavailability of pollutants, by means of adsorption and desorption from soil particles and transport. As a result, native microorganisms improve bioavailability by enlarging their external surface areas and by developing high fractal structures, such as hyphae [38], which increase the organisms contact with pollutants. The LbL coating is not only expected to improve bioavailability of pollutants due to its spiky structure and larger surface area; its high positive charge will also allow the biohybrid capsules to attach to soil particles, which has been shown to contribute to the bioavailability of soil-adsorbed atrazine [39].

Another reason for increased activity could be attributed to enhanced ATZ passive diffusion intracellularly. It was recently shown that the cell envelope of the Gram-negative *Polaromonas* sp. Nea-C, which is similar in structure to *E. coli*, acts as a barrier for ATZ biodegradation due to passive cell wall permeation [40]. The amine groups of polyDADMAC can disrupt the configuration of the outer membrane leading to increased permeability and at times bacteriostasis [41,42,43]. Therefore, the contaminant flux across the penetrated bacterial membrane/capsule interface is greater than that of intact bacterial membranes in free cells. This can also explain the advantage of the polyDADMAC capsules compared to chitosan and HPVP-*co*-S, as the high charge density and reactivity of quaternary amines is expected to promote higher membrane disruption [44].

When testing activity as a function of polyDADMAC/PSS layers (Figure 3d), it was visible that the intercellular ATZ flux decreased as the number of layers coating the cell increased, resulting in maximal biodegrading efficiency with one layer of the positive polycation. This is because the first layer of polyDADMAC damages the bacterial cell envelope resulting in high diffusion of ATZ, while the additional layers form a tight surrounding which hinders substrate diffusion. Similar observations were made by Sakkos et al. (2019) when coating of *E. coli* cells was performed with PolyDADMAC and silica alkoxide [20].

In addition, 1–3-layer capsules showed a lower polydispersity index (PDI) compared to 4–7 layer capsules, indicating good dispersion of 1–3 layered coated cells. A low PDI (<0.3) is typically a measurement of a sample containing a monodisperse population of particles, while a higher PDI (>0.7) is indicative of a highly polydispersed sample with multiple particle size populations [45]. The average PDI of 1–3 layers was 0.262 ± 0.13 while that of 4–7 layers was 0.848 ± 0.09. The PDI of FCs was lower than coated cells, 0.121 ± 0.04. Due to all these observations further in-depth characterization and activity assays were performed with the three layers polyDADMAC/PSS coated bacteria only.

### 3.3. Viability and Activity Assays

**Figure 4:**
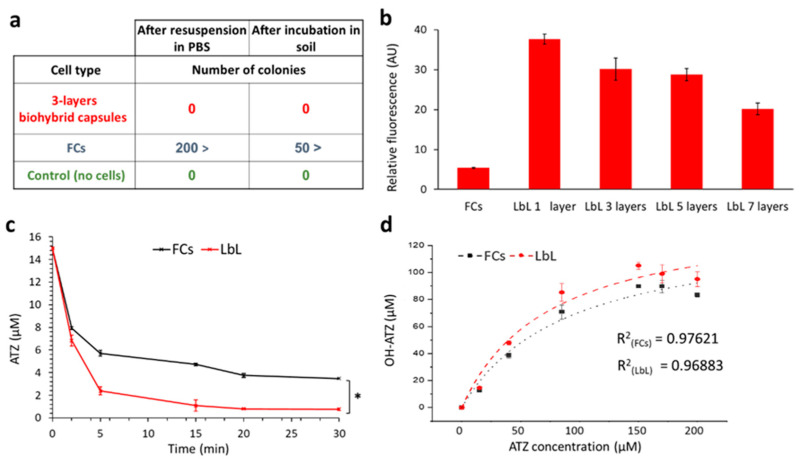
(**a**) Colony formation assay post resuspension in PBS and post incubation in soil (**b**) Relative fluorescence intensity in arbitrary units (**c**) Concentration of ATZ in the solution over time (initial concentration of 15 µM) (**d**) OH-ATZ formation at different initial ATZ concentrations. * Indicates significant difference between the groups (*p* < 0.05).

To further examine their potential as improved remediating agents, we focused on the ability of the coated cells to biodegrade ATZ without undesired proliferation. The AtzA enzyme is Fe(II)-dependent, operating without any other internal or external co-factors, thus the *E. coli* cells can be rendered non-viable yet remain active [46,47]. This is an important aspect in bioaugmentation; first, it can prevent the ecological stress associated with introducing non-indigenous populations into the well-defined natural hierarchy of soil microorganisms [48,49]. Also, it allows for a stable and continuous application of whole-cell biocatalysts, under undesired environmental conditions (salinity, temperature, availability of nutrients, etc.) that hinder microbial growth [50]. For this, the viability of the 3-layers PolyDADMAC/PSS biohybrid capsules was estimated by a colony formation assay, with plating done on solid LB medium with chloramphenicol and, in comparison to free cells. Plating was performed either immediately after the encapsulation process (resuspension in PBS) or after one week of incubation in sterile moistened soil, as described in Figure 4a. In addition, we performed a dead/live assay using propidium iodide (Figure 4b). PI is a cell-impermeant red-fluorescent stain that only labels cells with damaged cellular membranes. All the biohybrid capsules, regardless of the number of layers, showed high PI fluorescence intensity compared to the free cells, reinforcing the premise that polyDADMAC compromises the bacterial cell envelope facilitating diffusion of small organic molecules. In addition, combined with the colony formation assays, the dead/live viability assay further confirmed that encapsulated *E. coli* cells are non-vital.

Activity assays showed that despite their non-viability, the coating did not diminish the intracellular AtzA activity. Enzymatic kinetics experiment (Figure 4c) demonstrated that the 3-layers PolyDADMAC/PSS biohybrid capsules biodegraded 15 µM of ATZ more rapidly than free cells. In addition, concentration dependent assays (Figure 4d), were used to describe ATZ biodegradation according to a Michaelis−Menten-type kinetic model. Both assays demonstrated the superiority of the LbL coated bacteria over FCs in biodegrading ATZ. The *K_M_* value of FCs was higher than the *K_M_* of the 3-layers polyDADMAC/PSS biohybrid capsules (89.12 ± 29.48 µM and 74.51 ± 26.82 µM, respectively), while both were lower than previously reported free AtzA enzyme [25]. However, our findings are incomparable to previously reported values, including *V_max_* and *Kcat*, because whole cells do not encompass similar enzyme concentrations nor comparable molecular crowding [51]. Overall, the biohybrid capsules containing *E. coli* overexpressing AtzA, demonstrated strong biodegradation activity over the range of ATZ concentrations tested (1 to 20 mg∙L^−1^).

### 3.4. Morphology of the LbL Coated Cells

**Figure 5:**
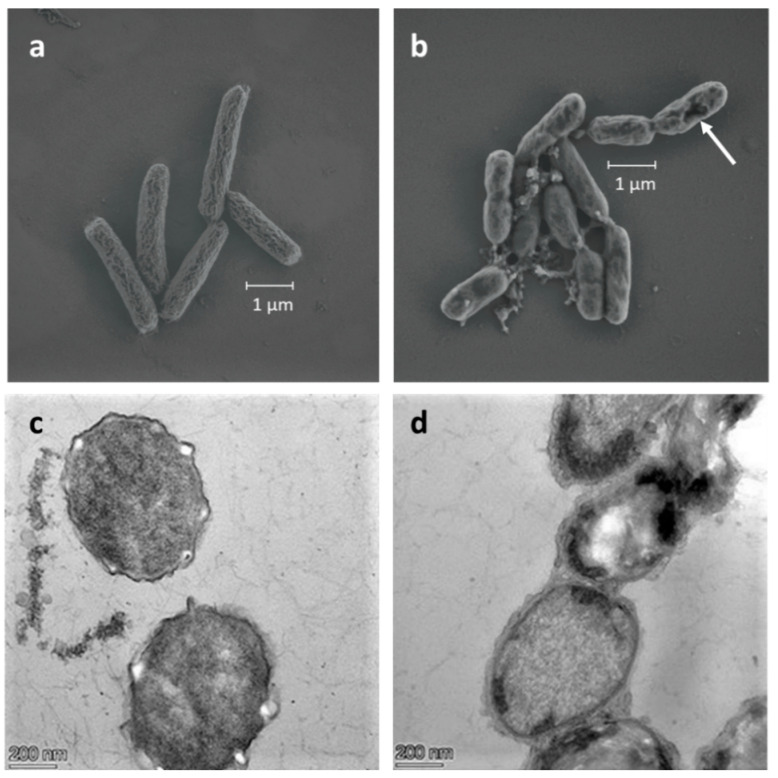
(**a**) SEM images of free *E. coli* cells and (**b**) 3-layers LbL biohybrid capsules. Arrows indicate damage seen in bacterial envelope. (**c**) TEM cross-section images of free *E. coli* cells and (**d**) 3-layers LbL biohybrid capsules.

Scanning electron micrographs of the 3-layers LbL coated cells, compared to FCs, are presented in Figure 5a,b. FCs exhibited a typical rod-like shape, whereas coated cells were less elongated and more rounded. The bio-hybrid capsules also showed some irregular fragments and loss of membrane integrity, similar to SEM images taken by Ansari et al., corroborating the speculated damage caused to the bacterial envelope by polyDADMAC [52]. Cross-sectional TEM images of the LBL bio-hybrid capsules revealed the presence of a thin, floccose layer, which is heterogeneous in shape and clearly different than the darken bacterial envelope of FCs (Figure 4c,d).

In addition to differences in the morphology of the bacterial envelopes, TEM images also revealed interior damages to the cells coated by the LbL polymer film. Healthy *E. coli* cells exhibit a homogeneous interior material distribution, which corresponds to proteins and DNA molecules, as seen in the free cell in Figure 4c. In the coated cells, however, a couple of dark, aggregated zones are seen, instead of a uniform cytoplasmic density. This lack of cytoplasmic content is representative of ‘ghost cells’—damaged cells that have undergone apoptosis [53]. These microscopic images further confirm the coating and the nonviable state of LbL-encapsulated cells.

### 3.5. Removal of ATZ from Soil

**Figure 6:**
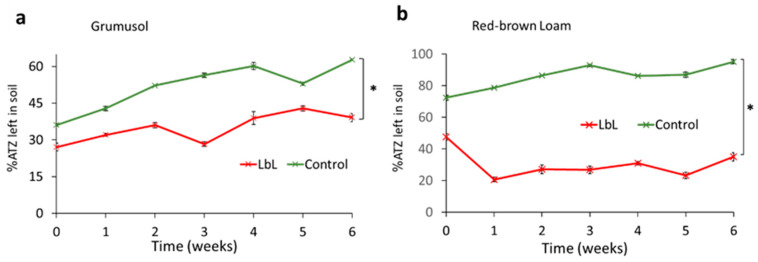
Removal of ATZ from contaminated soil over the course of 6 weeks: (**a**) Grumusol and (**b**) red-brown loam. The control measures ATZ concentrations in the soil without the addition of ATZ-degrading bacteria. * Indicates significant difference between the groups (*p* < 0.05).

To resemble a more realistic and systematic setting for bioremediation of ATZ, the 3-layered LbL biohybrid capsules were immobilized (by electrostatic interactions between the positively charged capsules and the negatively charged soil) onto two sterile, moistened soil samples, a clay rich Grumusol and a red-brown loam, which is low in clay content. The removal efficiencies of ATZ from spiked soils at 1–6 weeks of incubation are presented in Figure 6a,b (with time 0 being 30 min post incubation in soil). The bioremediation by biohybrid capsules was more successful in red-brown loam, with more than 84% accumulated total degradation of ATZ (calculated by mass balance with ATZ and OH-ATZ extraction in methanol after 6 weeks). The removal percentage was lower in clay-rich grumusol, with a total of ~69% accumulated degradation. These results are typical for the two soil types, since grumusol can retain organic pollutants via chemical adsorption to its clay and organic matter, whereas red-brown loam promotes higher mobility of pollutants. Thus, ATZ bioavailability is lower in clay-rich soils. In both soils, there was no significant difference (<0.05%) between LbL coated cells and FCs (data not shown in graphs). This could be attributed to the fact that FCs were able to proliferate in the soil as indicated by colony formation assays, therefore increasing the number of biodegrading agents over time. However, the non-viable biohybrid capsules maintained their biodegrading activity for 6 weeks, exhibiting stability of the AtzA enzyme and exceptional durability of the original capsules. Further investigation could include a more thorough soil analysis, such as an actual mimicking of a permeability barrier platform, to confirm suitability of the biohybrid capsules to existing remediation strategies [54]. These results suggest that these biohybrid LbL capsules possess high pollutant biodegradation ability unrelated to the increase in cell count post incubation in soil. Rather, the capsules can provide the AtzA enzyme with a protected, well-suited microenvironment, which allows the substrates and products to move freely in and out through the capsule.

## 4. Discussion

Herbicide-degrading bacteria were successfully coated with a thin polymeric casing via an LbL approach. Three cationic polymers were initially examined, PolyDADMAC, chitosan and H-PVP-*co*-S. All three polycation/PSS LbL coatings were successfully performed on the ATZ-degrading bacteria, yet only the polyDADMAC/PSS coating resulted in improved ATZ degradation rates. From the available data and the supporting literature, we suggest that polyDADMAC damages the bacterial membrane increasing diffusion rates into the cell and promoting degradation, whereas the other polymers create a diffusional barrier. The polyDADMAC-based bio-capsules were highly reactive, non-viable and carried a positive net charge to enable good adhesion to soil particles. Morphology observation with SEM and TEM confirmed the LbL layering, which led to bacterial envelope damage and subsequent death of cells. Still, activity remained high and rapid, as indicated by the kinetic and concentration activity assays. The biohybrid capsules provided an advantageous microenvironment suitable for prolonged enzymatic activity, which lasted for 6 weeks in polluted soil, and enhanced biodegradation by increasing specific surface area and enabling adequate flow of ATZ and OH-ATZ in and out of the cells. Improved biodegradation activity was noticeable in loamy soil, where pollutants are more prone to transport and therefore pose a higher risk to contaminate water sources [55]. Additional research is needed to evaluate the applicability of the coated cells in large scale systems, still, this study presents a proof-of-concept that such LbL capsules can be advantageous in complex environments. The versatility offered by the LbL encapsulation technique suggests that similar encapsulation of bioremediating agents could be tailored and adjusted to remediate a wide range of organic pollutants and can potentially be applied as an ecologically sustainable whole-cell enzyme delivery platforms for bioremediation and/or biocatalysis.
